# [Expression of Concern] Expression of L1 protein correlates with cluster of differentiation 24 and integrin β1 expression in gastrointestinal stromal tumors

**DOI:** 10.3892/ol.2026.15902

**Published:** 2026-10-12

**Authors:** Yue Du, Haihong Zhang, Zhongmin Jiang, Guowei Huang, Wenli Lu, Hesheng Wang

Oncol Lett 9: 2595–2602, 2015; DOI: 10.3892/ol.2015.3096

Following the publication of this paper, it was drawn to the Editor's attention by a concerned reader that, regarding the immunohistochemical images shown in Fig. 1 on p. 2599, the ‘L1/Grade 3’ and ‘Integrin β1/Grade 2’ data panels contained an overlapping section of data (albeit with different sizing, and turned through 180° relative to the other panel), suggesting that these images had been derived from the same original source, even though the results from differently performed experiments were intended to have been portrayed.

The authors have been contacted by the Editorial Office to offer an explanation for the apparently erroneous assembly of the data in Fig. 1, and we are awaiting their response. Owing to the fact that the Editorial Office has been made aware of potential issues surrounding the scientific integrity of this paper, we are issuing an Expression of Concern to notify readers of this potential problem while the Editorial Office continues to investigate this matter further.

